# Cognitive impairment in schizophrenia: relationships with cortical thickness in fronto-temporal regions, and dissociability from symptom severity

**DOI:** 10.1038/s41537-021-00149-0

**Published:** 2021-03-18

**Authors:** Erkan Alkan, Geoff Davies, Simon L. Evans

**Affiliations:** 1 Faculty of Health and Medical Sciences, University of Surrey, Guildford, Surrey UK; 2 Brighton & Sussex Medical School/Sussex Partnership NHS Foundation Trust, Sussex, UK

**Keywords:** Schizophrenia, Biomarkers

## Abstract

Cognitive impairments are a core and persistent characteristic of schizophrenia with implications for daily functioning. These show only limited response to antipsychotic treatment and their neural basis is not well characterised. Previous studies point to relationships between cortical thickness and cognitive performance in fronto-temporal brain regions in schizophrenia patients (SZH). There is also evidence that these relationships might be independent of symptom severity, suggesting dissociable disease processes. We set out to explore these possibilities in a sample of 70 SZH and 72 age and gender-matched healthy controls (provided by the Center of Biomedical Research Excellence (COBRE)). Cortical thickness within fronto-temporal regions implicated by previous work was considered in relation to performance across various cognitive domains (from the MATRICS Cognitive Battery). Compared to controls, SZH had thinner cortices across most fronto-temporal regions and significantly lower performance on all cognitive domains. Robust relationships with cortical thickness were found: visual learning and attention performance correlated with bilateral superior and middle frontal thickness in SZH only. Correlations between attention performance and right transverse temporal thickness were also specific to SZH. Findings point to the importance of these regions for cognitive performance in SZH, possibly reflecting compensatory processes and/or aberrant connectivity. No links to symptom severity were observed in these regions, suggesting these relationships are dissociable from underlying psychotic symptomology. Findings enhance understanding of the brain structural underpinnings and possible aetiology of cognitive impairment in SZH.

## Introduction

Schizophrenia is a complex disorder that includes widespread neurocognitive^1,2^ and neuroanatomical impairments^3,4^. Studies have shown that compared to healthy controls, schizophrenia patients (SZH) have impaired cognitive performance across all cognitive domains including processing speed^5^, attention and vigilance^6^, working memory^7^, verbal learning^8^, visual learning^9^, reasoning/problem solving^9^, and social cognition^10^. Impaired functioning is a hallmark of SZH and cognitive deficits have been shown to be closely tied to functional outcome in SZH^11–13^. However, cognitive impairments are largely unresponsive to pharmacological therapy^14^. These cognitive deficits are also observed in first episode psychosis samples suggesting they are not due to exposure to neuroleptic medication^13,15^. Cognitive training also has limited efficacy in certain domains. SZH show very limited response to perceptual training, for example, and while executive function training does have positive effects on neurocognition and functioning, these take several months to become apparent^16^. Developing a better understanding of the neuroanatomical basis of cognitive impairment in SZH could explain why this is so, and help inform treatment approaches.

Reduced cortical thickness has been consistently demonstrated in SZH^17^, including first episode psychosis^18^. The meta-analysis by van Erp et al.^17^, found evidence for widespread cortical thinning; effect sizes were largest in frontal and temporal regions. Strong cortico-cortical connectivity between affected regions has been shown and thus thinning appears to occur within an interconnected network^19^. While seemingly relatively stable and not linked to the duration of illness^20,21^, these morphological abnormalities have been linked to symptomology albeit inconsistently. Associations between positive symptom severity and thinning in frontal and temporal regions have been identified in SZH^22,23^ and first episode patients^21^; associations with negative symptoms seem less reliable. Abnormalities also link to the degree of cognitive impairment. A cognitively relatively intact subgroup of SZH seems to exist^24^ with less pronounced cortical thinning^25,26^; conversely, clustering patients according to cortical thickness patterns differentiate those with greater impairment^27^. The degree to which such neural correlates are attributable to cognitive impairment or to psychotic processes remains unclear, however. Some studies suggest that cognitively near-normal SZH show minimal cortical thinning compared to controls^25,28^ while others suggest otherwise^29,30^. Thus the role and significance of cortical thinning remain uncertain. Clarifying whether it associates mostly with cognitive impairment, or is attributable to a more general disease process, is important. If thinning relates specifically to cognitive impairment but not symptomology, this dissociability would point to separate disease pathologies and suggest that the core neurobiology of psychosis lies elsewhere, such as in subcortical nuclei^31^ and/or white matter pathways^32^. Thus, disentangling these relationships is important to enhance knowledge of the underlying neurobiology.

Studies into cognition-thickness relationships are informative in this regard. Limited data is available, but studies suggest relationships that are absent and/or specific to SZH within specific fronto-temporal regions^29,33,34^. Across various cognitive domains, Hanford et al.^29^ found cognition-thickness relationships in left superior temporal and right middle frontal that were present in controls but lacking in SZH, whereas left superior frontal thickness correlated positively with cognitive performance in SZH only, possibly indicating compensatory processes. Frontal cortex was also implicated by Oertel-Knöchel et al.^35^ who found cortical thickness in bilateral inferior frontal gyrus correlated with subjective cognitive dysfunction in SZH, but not in controls.

Temporal effects have also been shown. Hartberg et al.^34^ identified correlations between processing speed and thickness in left middle temporal and left transverse temporal that were present in SZH but not controls. Ehrlich et al.^36^ found that working memory performance in SZH correlated with thickness in right middle and right superior temporal gyrus (STG), rather than lateral prefrontal cortex as seen in controls. Edgar et al.^33^ focused only on cortical thickness in bilateral STG and prefrontal cortex: in SZH (but not in controls), thinning in left and right STG was associated with poorer attention performance on the Continuous Performance Test. STG included transverse temporal in these latter 2 studies. Finally, Pan et al.^27^ employed a clustering approach to categorise SZH with pronounced cortical thinning; this was evident across fronto-temporal regions, and this group showed greater working memory impairment.

Although studies are few and results are somewhat heterogeneous, evidence does suggest that there exist cognition-thickness relationships within temporal and frontal regions that are present in SZH but not in controls. However, some of the aforementioned studies only included a restricted range of cognitive domains. Here, we investigated links between cortical thickness and cognition across a broad range of cognitive domains using the standardised MCCB task battery, in a sample of seventy patients and controls. While Hanford et al.^29^ was also comprehensive in the MCCB domains included, the sample size employed here is larger. Whether cognitive-thickness relationships are dissociable from the degree of positive and negative symptomology also remains uncertain as few studies have explored this explicitly: here we compared correlations between thickness, cognition, and symptoms to address this question. Thus the current study aimed to better characterise relationships between cortical thickness and cognitive performance in SZH, and also interrogate whether these relationships are dissociable from links to symptom severity.

First, to characterise differences between SZH and controls, we assessed between-group differences in cognitive performance in six key domains (attention, working memory, reasoning/problem solving, processing speed, visual learning, and verbal learning). Overall composite cognitive scores were also considered. All measures were drawn from the Measurement and Treatment Research to Improve Cognition (MATRICS) Consensus Cognitive Battery. Cortical thickness within the regions of interest (ROIs, comprising regions implicated by previous studies) was also compared between-groups. Associations between domain scores and thickness were then assessed separately for SZH and controls, and correlations were compared between-groups to identify abnormal structure-function relationships in SZH. Associations between thickness and symptomology were also assessed in SZH to assess whether thickness within the ROIs was associated with symptom severity. Also, regression models investigated the extent to which variance in cognitive performance could be explained by symptomology, medication use, and other relevant variables. Based on previous findings we hypothesized lower cognitive performance on all cognitive domains, and thinner cortex in all ROIs, in SZH compared to healthy controls. We also expected to see thickness-cognition relationships in SZH that differ significantly from those present in healthy controls. Finally, we expected these thickness-cognition relationships in SZH to be at least partially dissociable from relationships between thickness and symptomology.

## Results

### Demographic data of the study sample

Means and standard deviations for all demographic data are reported in Table 1. No statistically significant differences were observed between SZH and healthy control groups on age and gender (all *p* values > 0.05). Healthy controls had a significantly higher level of education (M = 13.78, SD = 1.55) compared to SZH (M = 12.71, SD = 1.78, *p* < 0.001).

**Table 1:** Demographic characteristics of the sample.

	All Subjects (*N* = 142)	Healthy Controls (*N* = 72)	SZH (*N* = 70)	*p*
Age, M (SD)	38.01 (12.64)	38.44 (11.93)	37.56 (13.40)	0.677^a^
Female (N, %)	31 (21.8%)	18 (25%)	13 (18.6%)	0.354^b^
Education (years) (SD)	13.25 (1.74)	13.78 (1.55)	12.71 (1.78)	<0.001^a^
Smoking status (Smokers)	39 (27.7%)	14 (19.7%)	25 (35.7%)	0.053^b^
Medication (olanzapine equivalent mg/day) (SD)	N/A	N/A	21.67 (37.35)	N/A
PANSS (7 item) positive symptoms (mean) (SD)	N/A	N/A	15.25 (4.86)	N/A
PANSS (16 item) general psychopathology (mean) (SD)	N/A	N/A	29.19 (8.74)	N/A
PANSS (7 item) negative symptoms (mean) (SD)	N/A	N/A	14.78 (5.09)	N/A

*SZH* Schizophrenia, *SD* standard deviation, *PANSS* positive and negative syndrome scale, *N/A* not applicable.

^a^Independent sample *t* test.

^b^Pearson chi-Square.

### Neurocognitive performance of the study sample

Means and standard deviations for all MATRICS domain scores are reported in Table 2. ANCOVAs (age, gender, and education as covariates) showed that SZH had significantly lower performance on all MATRICS domain scores. All of these differences remained significant after correction for multiple comparisons (*p* < 0.05, FDR corrected).

**Table 2:** Neurocognitive performance differences (age, gender and education as covariates).

	Mean Scores (SD)		ANCOVA		
Tests	HC	SZH	F	*P*_*unadjusted*_	*P*_*FDR*_
MATRICS domain processing speed	53.625 (8.51)	35.956 (11.22)	93.049**	<0.001	<0.001
MATRICS domain working memory	48.681 (7.57)	39.414 (12.27)	22.063**	<0.001	<0.001
MATRICS domain visual learning test	46.194 (10.01)	35.886 (12.03)	26.484**	<0.001	<0.001
MATRICS domain attention/vigilance	50.030 (8.66)	37.243 (13.33)	38.071**	<0.001	<0.001
MATRICS domain reasoning/problem solving	55.314 (8.42)	44.338 (10.65)	35.341**	<0.001	<0.001
MATRICS domain verbal learning	45.390 (6.70)	38.542 (8.47)	20.881**	<.0001	<0.001
MATRICS domain overall composite scores	50.323 (6.58)	32.182 (12.41)	88.309**	<0.001	<0.001

**FDR corrected *p* < 0.05.

*HC* healthy controls, *SZH* Schizophrenia.

### MRI–Cortical thickness differences between SZH and controls

Between-group cortical thickness differences were assessed using ANCOVA with age and gender as covariates.

After FDR correction for multiple comparisons, thinner cortices were observed in SZH within superior frontal (bilateral), middle frontal, inferior frontal, superior temporal (bilateral), and middle temporal (*p* < .05, FDR corrected) (Table 3). For between-group differences in other brain regions that were not included in our ROIs, see Supplementary Table 5.

**Table 3:** Cortical Thickness, group comparisons (ANCOVA, age, and gender as covariates).

	Mean thickness				
Regions	HC	SZH	*F*	*P*_*unadjusted*_	*P*_*FDR*_
Right superior frontal	2.68	2.62	12.337**	0.001	0.002
Left superior frontal	2.72	2.65	12.411**	0.001	0.002
Middle frontal	2.47	2.42	12.947**	<0.001	0.002
Inferior frontal	2.61	2.51	35.165**	<0.001	0.002
Left superior temporal	2.79	2.73	7.840**	0.006	0.008
Right superior temporal	2.82	2.74	10.802**	0.001	0.002
Middle temporal	2.88	2.82	12.356**	0.001	0.002
Right transverse temporal	2.40	2.35	2.177	0.142	0.160
Left transverse temporal	2.32	2.30	0.362	0.549	0.549

**FDR corrected *p* < 0.05.

*HC* healthy controls, *SZH* Schizophrenia.

### Multiple regression analysis

Overall, only PANSS negative and medication use explained variance in cognitive performance, duration of illness, gender, PANSS positive, and education were not significant predictors in any of the models. PANSS negative (*p* = 0.002) and medication (*p* = 0.021) were significant predictors of processing speed. Medication use was the only predictor of attention/vigilance scores (*p* = 0.040). PANSS negative score was the only predictor of reasoning/problem-solving scores (*p* = 0.021). Models did not explain significant variance in working memory or visual memory performance. PANSS negative (*p* = 0.011) and medication (*p* = 0.031) were significant predictors of overall composite scores (Supplementary Table 1).

### Correlations between MATRICS domain scores and cortical thickness

Partial correlation analyses (controlling for age and education) investigated relationships between neurocognitive performance and cortical thickness within the ROIs. Correlations were assessed separately for each group and compared using a percentile bootstrap method.

Processing speed: scores in SZH positively correlated with right transverse temporal thickness (uncorrected *p* = 0.023) although this was not significant after FDR correction. No associations were seen in healthy controls (all *p* values > 0.05) (Table 4). A significant difference in correlations between groups was identified for this region, as the confidence intervals for the bootstrap distribution of the differences between correlation coefficients did not overlap with zero (95% CI: 0.29, 0.41).

**Table 4:** Correlations between MATRICS domains (processing speed and attention/vigilance) and cortical thickness, by group (controlling for age and education).

	Processing speed						Attention/Vigilance					
Cortical Thickness	Controls			SZH			Controls			SZH		
	*r*	*P*_*unadjusted*_	*P*_*FDR*_	*r*	*P*_*unadjusted*_	*P*_*FDR*_	*r*	*P*_*unadjusted*_	*P*_*FDR*_	*r*	*P*_*unadjusted*_	*P*_*FDR*_
Right superior frontal	−0.053	0.660	0.950	0.172	0.165	0.358	−0.079	0.533	0.887	0.311**	0.010	0.030
Left superior frontal	0.045	0.714	0.950	0.159	0.199	0.358	−0.069	0.586	0.887	0.321**	0.008	0.030
Middle frontal	0.046	0.708	0.950	0.210	0.089	0.267	0.064	0.615	0.887	0.273	0.024	0.054
Inferior frontal	−0.029	0.812	0.950	0.211	0.086	0.267	−0.004	0.976	0.976	0.217	0.076	0.114
Left superior temporal	−0.008	0.950	0.950	0.075	0.546	0.546	−0.100	0.426	0.887	0.195	0.111	0.143
Right superior temporal	0.012	0.921	0.950	0.111	0.373	0.468	−0.024	0.847	0.953	0.245	0.044	0.079
Middle temporal	−0.113	0.353	0.950	0.107	0.388	0.468	−0.053	0.676	0.887	0.169	0.168	0.168
Right transverse temporal	−0.173	0.153	0.950	0.277	0.023	0.207	−0.050	0.690	0.887	0.333**	0.006	0.030
Left transverse temporal	−0.127	0.295	0.950	0.101	0.416	0.468	−0.194	0.121	0.887	0.180	0.143	0.161

**FDR corrected *p* < 0.05.

*SZH* Schizophrenia.

**Fig. 1:**
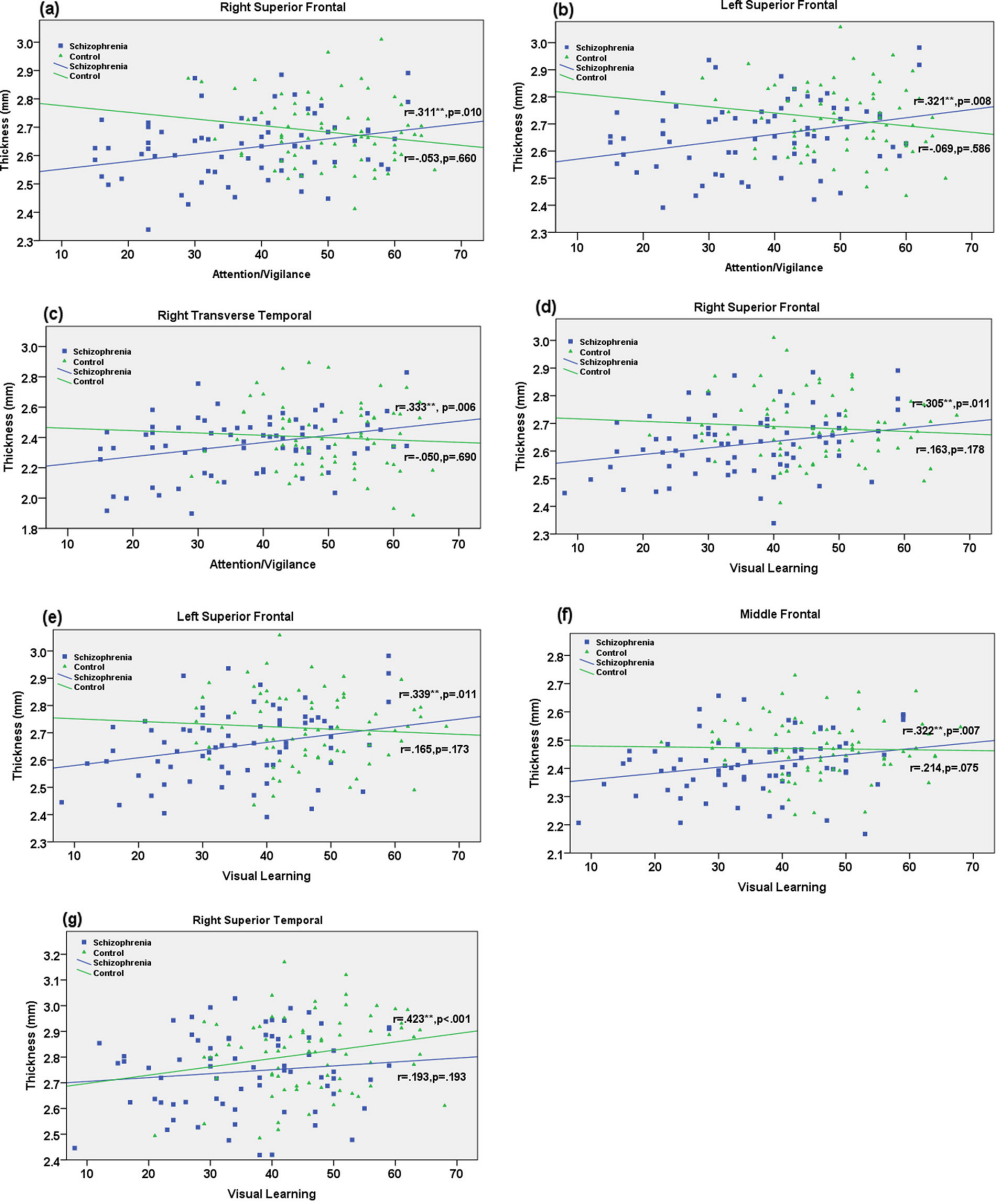
Correlations between cortical thickness and cognitive performance scores. Scatter plots demonstrating significant positive relationships between cognitive domains and cortical thickness (mm), adjusted for age and gender, in SZH and controls. **a** Larger cortical thickness in right superior frontal related to the better attention/vigilance performance in SZH (*r* = 0.311, *p* = 0.010). **b** Larger thickness in left superior frontal related to the better attention/vigilance performance in SZH (*r* = 0.321, *p* = 0.008). **c** Larger thickness in right transverse temporal thickness related to the better attention/vigilance performance in SZH (*r* = 0.333, *p* = 0.006). **d** Larger thickness in right superior frontal positively correlated with better visual learning performance in SZH (*r* = 0.305, *p* = 0.011). **e** Larger left superior frontal positively correlated with better visual learning performance in SZH (*r* = 0.339, *p* = 0.011). **f** Larger middle frontal thickness positively correlated with better visual learning performance in SZH (*r* = 0.322, *p* = 0.007). **g** Larger right superior temporal thickness positively correlated with visual learning in healthy controls (*r* = 0.423, *p* < 0.001).

Attention and Vigilance: Scores in SZH positively correlated with thickness in left and right superior frontal (see Figs. 1a, 1b) and right transverse temporal thickness (see Fig. 1c), and these relationships were significant after FDR correction. A positive correlation with middle frontal was observed in SZH but was only present at trend (*p* = 0.054) after FDR correction. A positive correlation with right superior temporal was observed in SZH (uncorrected *p* = 0.044) but did not survive FDR correction. No associations were seen in healthy controls (Table 4). Between-groups, significant differences in correlations was found for left (95% CI: 0.32, 0.43), and right (95% CI: 0.34, 0.45) superior frontal, and right transverse temporal (95% CI: 0.25, 0.37).

Visual learning: scores in SZH positively correlated with thickness in left and right superior frontal (see Fig. 1d, 1e) and middle frontal (see Fig. 1f) and these were significant after FDR correction. A relationship with middle temporal was observed (*p* = 0.039) which did not survive FDR correction; a strong uncorrected trend (*p* = 0.053) was seen in left transverse temporal. In controls, visual learning scores positively correlated with thickness in right superior temporal (see Fig. 1g) which survived FDR correction, significant uncorrected relationships were present in left superior and middle temporal although were only seen at trend-level after FDR-correction (Table 5). No difference in correlations between groups was found for any of these regions.

**Table 5:** Correlations between MATRICS domains (visual learning and composite scores) and cortical thickness, by group (controlling for age and education).

	Visual learning						Composite scores					
Cortical thickness	Controls			SZH			Controls			SZH		
	*r*	*P*_*unadjusted*_	*P*_*FDR*_	*r*	*P*_*unadjusted*_	*P*_*FDR*_	*r*	*P*_*unadjusted*_	*P*_*FDR*_	*r*	*P*_*unadjusted*_	*P*_*FDR*_
Right superior frontal	0.163	0.178	0.200	0.305**	0.011	0.033	0.029	0.819	0.921	0.205	0.104	0.187
Left superior frontal	0.165	0.173	0.200	0.339**	0.005	0.032	0.114	0.373	0.753	0.242	0.054	0.162
Middle frontal	0.214	0.075	0.169	0.322**	0.007	0.032	0.074	0.563	0.753	0.243	0.054	0.162
Inferior frontal	0.199	0.099	0.178	0.223	0.068	0.087	0.112	0.383	0.753	0.207	0.100	0.187
Left superior temporal	0.273	0.022	0.066	0.191	0.119	0.119	0.094	0.466	0.753	0.144	0.258	0.332
Right superior temporal	0.423**	<0.001	<0.001	0.193	0.114	0.119	0.235	0.063	0.545	0.114	0.369	0.415
Middle temporal	0.300	0.012	0.054	0.251	0.039	0.087	0.197	0.121	0.545	0.146	0.251	0.332
Right transverse temporal	0.180	0.137	0.200	0.224	0.066	0.087	-0.009	0.946	0.946	0.264	0.035	0.162
Left transverse temporal	0.145	0.232	0.232	0.235	0.053	0.087	-0.070	0.586	0.753	0.068	0.592	0.592

**FDR corrected *p* < 0.05.

*SZH* Schizophrenia.

Working memory: No associations were seen either in healthy controls or SZH (Supplementary Table 2).

Reasoning/Problem Solving: No associations were seen either in healthy controls or SZH (Supplementary Table 2).

Verbal learning: No associations were seen either in healthy controls or SZH (Supplementary Table 3).

Composite scores: In SZH, there was evidence of a positive relationship between composite scores and thickness in right transverse temporal (*p* = 0.035) although this did not survive FDR correction. Strong uncorrected positive trends were seen in middle frontal (*p* = 0.054) and left superior frontal (*p* = 0.054). No associations were seen in healthy controls (Table 5). Between-group differences in correlations were observed for right transverse temporal (95% CI: 0.25, 0.37).

### Correlations between symptom severity and cortical thickness

Partial correlation analyses (controlling for age, education, and olanzapine equivalent) were performed to investigate relationships between symptom severity and cortical thickness within the ROIs.

Correlates of positive symptoms: Negative correlations were observed with inferior frontal thickness (uncorrected *p* = 0.017) and left superior temporal thickness (uncorrected *p* = 0.041), although these were not significant after FDR correction. No other associations were observed (Supplementary Table 4).

Correlates of negative symptoms: No associations were observed (Supplementary Table 4).

## Discussion

The present study set out to investigate whether cortical thickness within frontal and temporal brain regions correlates with neurocognitive performance in SZH, whether these relationships differed from those of a healthy control group with similar age and gender composition, and whether these relationships were dissociable from those linked to symptom severity. As expected, significant group differences in cognitive performance were found with SZH showing poorer performance in all cognitive domains under study. The level of medication use explained significant variance in cognitive performance, in line with previous results^52^.

ROIs were selected based on previous work suggesting structure-function relationships particular to SZH might be present within specific fronto-temporal regions. As expected, significantly thinner cortex within the ROIs were evident in SZH, in superior, middle, and inferior frontal, and superior and middle temporal regions (but not transverse temporal) while controlling for age and gender. This is in line with the previous literature^53,54^. We focused on cortical thickness since brain volume measures are influenced by cortical surface area as well as thickness. Generally, surface area in SZH shows fewer links to symptomology and differs less against control groups^23^. Cortical thickness specifically reflects the density and size of neuronal cells and neuropil (axons + dendrites + glia)^55,56^. Postmortem studies in SZH point to reduced density of neuronal cells and neuropil, while the actual number of neurons appears to be unaffected^57,58^; cortical thickness measures might thus be more sensitive than volumetric comparisons.

In terms of links between symptom severity and thickness within the ROIs, positive symptoms correlated with thickness in inferior frontal and left superior temporal regions, but these did not survive FDR correction. Reduced inferior frontal volume^23,59^ and reduced bilateral superior temporal thickness^60^ have previously been linked to positive symptoms severity in SZH. No associations with negative symptoms were seen, in contrast to a large recent meta-analysis which pointed to associations in prefrontal regions^61^. However, links to negative symptoms are less robust in the literature, possibly reflecting a relatively greater contribution of other neural parameters and social influences to negative symptomology^23^.

Our main aim was to examine between-group differences in associations between cortical thickness and cognitive performance. Results revealed some robust relationships in SZH that were absent in controls. Better performance in attention/vigilance associated with thicker right transverse temporal and bilateral superior frontal in SZH, but not in controls. Processing speed scores in SZH also positively correlated with right transverse temporal thickness: although this did not survive FDR correction, the difference in correlations between groups was significant. Visual learning associated with thicker bilateral superior and middle frontal in SZH, in controls associations were seen with right superior temporal thickness but no between-group differences in correlations were found for this cognitive domain. No relationships were seen for working memory or reasoning/problem-solving. On the composite score, there was evidence of an (uncorrected) positive relationship in SZH with thickness in right transverse temporal; a significant between-group difference in correlations was observed for this region. Overall, results pointed to abnormal structure-function relations in SZH in superior frontal and right transverse temporal regions.

Attention/vigilance performance correlated with superior frontal cortex thickness in SZH only. Superior frontal cortex was seen to be thinner in the SZH group overall, when compared to controls. Reduced pyramidal layer thickness seems to be the major contributor to decreased frontal lobe thickness in SZH^62^. Pyramidal neurons function as output cells; loss of these likely affects intra- and inter-region connectivity with implications for advanced cognitive functioning. Aberrant pruning processes during development^63^ and reduced blood flow in the prefrontal cortex^62^ are likely contributing factors. Hanford et al.^29^ observed a positive relationship between superior frontal thickness and overall cognitive performance, in SZH only (also using the MATRICS battery, but in SZH/control groups that were matched on cognitive ability). Hanford et al.^29^ suggest this could reflect operation of compensatory processes in higher-performing SZH. Furthermore, enhanced functional connectivity of the left superior frontal gyrus has been put forward as a potential phenotypic marker for the disease: Ding et al.^64^ found increased functional connectivity specific to the left superior frontal gyrus in both drug-naive SZH and their unaffected siblings; connectivity values of this region could differentiate patients and siblings from controls with good sensitivity, and values did not correlate with symptom severity or illness duration suggesting it could represent a trait marker. However, Ding et al.^64^ did not assess links with cognition; the current findings suggest this is an important avenue to be explored.

Results pointed to a relationship between right transverse temporal thickness and attention/vigilance; relationships between right transverse temporal thickness, processing speed, and composite scores were present at an uncorrected threshold. These correlations were found to be significantly different between groups, suggesting SZH-specific relationships between right transverse temporal thickness and cognition. Previous studies identified relationships between thickness of right STG (incorporating transverse temporal) and attention^33^, and verbal processing/working memory recall^36^, these relationships were absent in controls and present bilaterally in Edgar et al.^33^. Hartberg et al.^34^ found a positive relationship between left transverse temporal thickness and processing speed only in SZH and not controls. Taken together, findings confirm the particular importance of temporal lobe thickness (and possibly transverse regions in particular) for cognitive function in SZH across multiple domains. There are various possibilities that could explain this. Firstly, decreased or reversed anatomical asymmetry (particularly in the temporal lobe) has been reported in SZH^65^ which could potentially contribute^33^, although here we found overall between-group differences were present in left and right superior, but not transverse, temporal cortices. Alternatively (or in addition) SZH could be engaging a larger network of cortical regions to compensate for prefrontal cortical dysfunction^66^. Altered connectivity and fronto-temporal network dysfunction (as discussed above) could be of importance in this. Given that we found SZH-specific relationships for attention/vigilance performance with both right transverse temporal and bilateral superior frontal thickness, this explanation is supported by the data. Further investigations, linking the findings reported here to aberrant fronto-temporal connectivity patterns, would be worthwhile.

Also worth noting are previous findings showing that volume loss in transverse temporal regions (Heschl’s gyrus, primary auditory cortex) correlates with the severity of auditory hallucinations in SZH. This correlation seems to be more robust with left transverse temporal^67,68^ but right-lateralised associations have also been reported^68,69^. More broadly, positive symptom severity has been seen to correlate with STG thinning in both hemispheres^61^ and could, therefore, be a contributing factor to structure-cognition relationships in STG^70^.

In the current study, regions showing links to cognitive performance did not overlap with those showing links to symptom severity. Indeed, although we found some association between positive symptoms and thinning in inferior frontal and left superior temporal regions, these did not survive multiple comparisons correction. This contrasts with the statistically robust relationships between thickness and cognition identified in the SZH group. This supports the suggestion that structure-cognition relationships in SZH are dissociable from symptomology, pointing to independent processes. Furthermore, regression models showed that PANSS positive symptoms did not explain variance in any cognitive domain, although PANSS negative did explain significant variance in processing speed, reasoning/problem solving, and overall composite scores. However, we cannot discount the possibility that the present study lacked the sensitivity to detect relationships with symptoms. Although larger than many previous investigations into structure-cognition relationships, the sample size was relatively modest compared to recent studies investigating links to symptoms: a greater number of participants might have led to the detection of more subtle differences. Moreover, even though the study sample well-matched on age and gender, lower levels of education in the SZH group might have affected results. However, we adjusted all analyses for education, and unmatched education level is common in the literature^6,33,71,72^. Sensitivity of the measures used might also be an issue. Previous work into structure-cognitive function relationships in SZH have yielded inconsistent results, and not all studies have found evidence that relationships differ between SZH and controls (e.g., Hartberg et al.^72^). We did not find any relationships between cortical thickness and reasoning/problem solving or working memory (in contrast with Ehrlich et al.^36^, who used different memory tasks): choice of methodology might be a factor. Other neuropsychological tests might be more sensitive to relationships between cortical structures and performance in these functional domains in SZH, and this is also true for the symptom measures. This is exemplified by Oertel-Knöchel et al.^35^ who found no relation between cortical thinning and PANSS scores, but a correlation between left STG thinning and hallucinations was revealed when the Revised Hallucination Scale was used instead. Also, some reports point to a tighter coupling between hippocampus volume and memory performance in SZH compared to controls^36^ but subcortical structures were not included in the present investigation. Finally, recent work has shown that global fractional anisotropy^73^ and genomic^74–77^ contributions to cognition overlap substantially between SZH and controls. The SZH-specific thickness-cognition relationships identified here should be considered, and explored further, in this context.

In conclusion, this study provides important details regarding cortical thickness-cognition links in SZH which remains an important but understudied field of investigation. SZH showed widespread cortical thinning across most of the fronto-temporal regions under study, and significantly impaired performance across all cognitive domains. Based on a highly reliable and validated cognitive battery, cortical thickness in bilateral superior frontal and right transverse temporal regions correlated positively with cognitive performance (particularly attention/vigilance) in patients but not controls. These findings accord with previous results suggesting that thickness in these regions are of specific importance for cognitive performance in SZH, possibly reflecting compensatory processes. On the other hand, no links to symptom severity were present in these regions, suggesting the structural underpinnings of cognitive deficits might be somewhat dissociable from that of symptom severity and could reflect separate disease processes. Delineating the neural bases of each are of importance in developing our understanding of the aetiology of this complex disease.

## Methods

### Subjects

The data for 70 SZH (18 females, aged between 19 and 65, mean age 37.56, see Table 1) and 72 age and gender-matched healthy controls (18 females, aged between 18 and 65, mean age 38.44, see Table 1) from the publicly-available dataset provided by the Center of Biomedical Research Excellence (COBRE, http://fcon_1000.projects.nitrc.org/indi/retro/cobre.html)^37–40^ were used for this study. The Positive and Negative Syndrome Scale (PANSS)^41^ was used to assess symptoms. Written informed consent was obtained before participation under the guidelines of the Institutional Review Board at the University New Mexico (UNM)^42^. All patients were receiving antipsychotic medication: doses at the time of MRI scan were converted to olanzapine equivalents (see Table 1).

Cognitive scores were screened and individuals with outliers in any domain were excluded based on Interquartile Range (IQR): criteria for exclusion were values below Q1-1.5 IQR or above Q3 + 1.5 IQR, box-and-whisker plots were used^78,79^ (also known as Tukey’s method^80,81^). After removing individuals with outliers the final sample consisted of 70 SZH (18 females, aged between 19 and 65, mean age 37.56, see Table 1) and 72 age and gender-matched healthy controls (18 females, aged between 18 and 65, mean age 38.44, see Table 1). Schizophrenia was diagnosed by a psychiatrist using the Structured Clinical Interview for Diagnostic and Statistical Manual of Mental Disorders, Fourth Revision (DSM-IV)^82^. The exclusion criteria were defined by the research centre as intellectually disability, head trauma with more than 5 minutes loss of consciousness, history of substance abuse or dependence within the previous 12 months, or neurological disorder.

The authors assert that all procedures contributing to this work comply with the ethical standards of the relevant national and institutional committees on human experimentation and with the Helsinki Declaration of 1975, as revised in 2008.

### MRI acquisition

The COBRE group collected raw structural imaging data with a Siemens TIM Trio 3 T scanner (Siemens Erlangen, Germany) using a 12-channel head coil. The details of the procedure are described elsewhere^43,44^, also see http://schizconnect.org/documentation#data_models. High-resolution T1-weighted structural images were acquired using an MPRAGE sequence with the following parameters: TR/TE/TI = 2530/[1.64, 3.5, 5.36, 7.22, 9.08]/900 ms, flip angle = 7°, FOV = 256 × 256 mm, Slab thickness = 176 mm, Matrix = 256x256x176, Voxel size =1x1x1 mm, Number of echos = 5, Pixel bandwidth =650 Hz, Total scan time = 6 min. All images were visually inspected, and then cortical reconstruction was performed using the Freesurfer 6.0.0 image analysis suite (http://surfer.nmr.mgh.harvard.edu). The cortical parcellation was based on the Destrieux atlas^45^.

The reconstruction pipeline employed by FreeSurfer includes intensity normalization, motion correction, and the exclusion of non-brain tissue was performed using a hybrid watershed/surface deformation procedure. Images are transformed to Talairach space and the subcortical white matter and deep grey matter structures are segmented^83,84^. Cortical thickness measurements were calculated using FreeSurfer’s ‘recon -all’ pipeline which calculates the average distance between the pial surface and the white/grey matter boundary for each region^85,86^.

### Cognitive measures

All participants completed the MATRICS (http://www.matricsinc.org/) battery which includes seven cognitive domains previously shown to be impaired in SZH, namely, working memory, attention/vigilance, verbal learning and memory, visual learning and memory, reasoning and problem solving, speed of processing, and social cognition^46–49^. Scores for each domain as well as a composite score -an average of the t-scores from cognitive domains- were converted to standardized T-scores by the COBRE group and our analyses were based on these. The duration of administration is ~70 min per person. Domain scores are a composite based on a variety of independent tests with satisfactory validity and reliability^47^. The current study considered MATRICS total scores, and scores from the following domains. The individual tests make up these domains are:

- Speed of Processing: Brief Assessment of Cognition in Schizophrenia (BACS): Symbol-Coding; Category Fluency test: Animal Naming, Trail Making Test: Part A (TMT-A),
- Attention/Vigilance: Continuous Performance Test-Identical Pairs (CPT-IP)
- Working Memory: Wechsler Memory Scale, 3rd Edition (WMS-III): Spatial Span, Letter-Number Sequencing (LNS)
- Visual Learning: Brief Visuospatial Memory Test-Revised (BVMT-R)
- Reasoning and Problem Solving: Neuropsychological Assessment Battery (NAB): Mazes
- Verbal Learning: Hopkins Verbal Learning Test–Revised

### Statistical analysis

IBM SPSS Statistics 21.0 was used to perform statistical analyses, p values are reported two-tailed and *p* < 0.05 was considered statistically significant. On demographic variables, groups were compared using independent sample *t* tests (for age and education), and a *χ*^2^ test (for gender). To test the cognitive performance differences, Analyses of covariance (ANCOVAs) were performed in which age, gender, and education level were included as covariates. For cortical thickness between-group comparisons, ANCOVA was used in which age and gender were included as covariates. Corrections for multiple comparisons were performed using the false discovery rate (FDR) with the Benjamini-Hochberg method^50^, and the significance level was set at 0.05.

To assess factors that explain variance in cognitive performance in the SZH group, a series of multiple regression models were constructed for each cognitive domain under study and the overall composite score. Duration of illness, gender, medication (olanzapine equivalent), education, PANSS positive, and PANSS negative were entered as independent variables and significance was defined as *p* < 0.05.

Partial correlations were performed for SZH and control group separately, with age and education as control variables. Follow-up analyses also included medication use as an additional covariate and are reported in Supplementary Tables 6 and 7. The ROIs encompassed a set of frontal and temporal regions where previous work has identified structure-cognition relationships in SZH that differ from those seen in controls. The frontal ROIs were: superior frontal (bilateral) and middle frontal (as shown by Hanford et al.^29^, measure: overall cognitive performance), inferior frontal (as shown by Oertel-Knöchel et al.^35^, measure: subjective cognitive dysfunction). Temporal ROIs were: transverse temporal (bilateral) (as shown by Hartberg et al.^34^, measure: processing speed), superior temporal (bilateral) (as shown by Ehrlich et al.^36^, measure: working memory; and Edgar et al.^33^, measure: attention) and middle temporal (as shown by Ehrlich et al.^36^, measure: working memory; and Hartberg et al.^34^, measure: processing speed). To control for multiple comparisons, FDR corrections were applied as above.

To test for differences in correlations between groups, a frequentist approach was taken using a percentile bootstrap method^51^. Between-group differences were assessed when a significant correlation was identified in one of the groups (FDR-corrected). Exploratory analyses also tested for between-group differences in correlations when a correlation was seen to be significant prior to FDR correction. Using a percentile bootstrap method with 5000 iterations, the distribution of bootstrap correlation differences is assessed to determine significance. A significant difference in correlations is inferred if the 95% percentile bootstrap confidence intervals do not overlap with zero. This analysis was performed in Matlab using code adapted from https://github.com/GRousselet/blog/tree/master/comp2dcorr and is more robust than other approaches^87^.

### Reporting summary

Further information on research design is available in the Nature Research Reporting Summary linked to this article.

## Supplementary information

Supplementary Information

Reporting Summary

## Data Availability

The dataset is freely available in the following website: http://fcon_1000.projects.nitrc.org/indi/retro/cobre.html.

## Code availability

The MATLAB code that used for statistical analyses is freely available in the following website: https://github.com/GRousselet/blog/tree/master/comp2dcorr.
