# Supplementary material for: Cognitive impairment in schizophrenia: relationships with cortical thickness in fronto-temporal regions, and dissociability from symptom severity
Source: NPJ Schizophr. 2021 Mar 18;7:20. doi: 10.1038/s41537-021-00149-0 (PMC7973472; doi:10.1038/s41537-021-00149-0)
Supplement: Supplementary file 1 — Supplementary Information [file 41537_2021_149_MOESM1_ESM.pdf]

Supplementary Table 1.

Results of Multiple Linear Regression Analysis for Variables Predicting MATRICs Domain scores in SZH

	Processing Speed		Attention/Vigilance		Working Memory		Visual Learning		Reasoning/Problem Solving		Composite Score	
Variable	***β***	***p***	***β***	***p***	***β***	***p***	***β***	***p***	***β***	***p***	***β***	***p***
Duration of Illness	.052	.665	-.021	.874	-.014	.917	.137	.298	-.073	.571	-.024	.850
Gender	-.009	.936	.015	.908	.039	.764	.046	.721	-.168	.191	.022	.865
Education	-.003	.978	-.006	.962	.052	.706	.061	.656	.142	.297	.076	.570
Olanzapine	-.283*	.021	-.267*	.040	-.219	.094	-.080	.536	-.129	.316	-.331**	.011
PANSS Negative	-.403**	.002	-.081	.541	-.144	.286	-.237	.081	-.314*	.021	-.287*	.031
PANSS Positive	-.042	.731	-.178	.172	-.133	.314	.055	.673	-.036	.785	-.049	.700

**p< .05. **p<.01*

Abbreviation: SZH, Schizophrenia

Supplementary Table 2.
Correlations Between MATRICS Domains and cortical thickness, by group (controlling for age and education)

	**Working Memory**						**Reasoning Problem Solving**					
Cortical Thickness	**Controls**			**SZH**			**Controls**			**SZH**		
	r	$P_{unadjusted}$	P_{FDR}	r	$P_{unadjusted}$	P_{FDR}	r	$P_{unadjusted}$	P_{FDR}	r	$P_{unadjusted}$	P_{FDR}
Right Superior Frontal	-0.061	0.617	0.981	0.013	0.918	0.998	-0.075	0.541	0.976	0.089	0.477	0.613
Left Superior Frontal	0.010	0.931	0.981	0.032	0.794	0.998	0.004	0.976	0.976	0.119	0.340	0.612
Middle Frontal	-0.009	0.942	0.981	0.095	0.439	0.998	0.030	0.807	0.976	0.200	0.107	0.321
Inferior Frontal	-0.047	0.700	0.981	0.049	0.694	0.998	0.096	0.434	0.976	0.213	0.087	0.321
Left Superior Temporal	-0.060	0.622	0.981	0.033	0.791	0.998	-0.006	0.961	0.976	0.144	0.249	0.560
Right Superior Temporal	0.049	0.684	0.981	0.002	0.988	0.998	0.123	0.318	0.976	0.091	0.465	0.613
Middle Temporal	-0.003	0.981	0.981	0.029	0.814	0.998	0.033	0.788	0.976	0.047	0.710	0.799
Right Transverse Temporal	-0.012	0.923	0.981	0.114	0.353	0.998	-0.049	0.689	0.976	0.227	0.067	0.321
Left Transverse Temporal	-0.252	0.035	0.315	0.000	0.998	0.998	0.090	0.467	0.976	0.010	0.939	0.939

*p<.05, **p<.01
Abbreviations. SZH, Schizophrenia

Supplementary Table 3.

Correlations Between verbal learning and cortical thickness (controlling for age and education)

Cortical Thickness	Controls			SZH		
	r	$P_{unadjusted}$	P_{FDR}	*r*	$P_{unadjusted}$	P_{FDR}
Right Superior Frontal	0.063	0.602	0.917	0.106	0.390	0.947
Left Superior Frontal	0.078	0.520	0.917	0.110	0.372	0.947
Middle Frontal	0.024	0.841	0.917	0.073	0.555	0.947
Inferior Frontal	-0.043	0.722	0.917	-0.018	0.885	0.947
Left Superior Temporal	-0.013	0.917	0.917	-0.081	0.513	0.947
Right Superior Temporal	0.029	0.811	0.917	-0.041	0.740	0.947
Middle Temporal	0.100	0.408	0.917	0.019	0.876	0.947
Right Transverse Temporal	0.018	0.881	0.917	0.187	0.126	0.947
Left Transverse Temporal	-0.074	0.545	0.917	0.008	0.947	0.947

**FDR corrected p<0.05

Abbreviations. SZH, Schizophrenia

Supplementary Table 4.

Correlations Between Symptom severity and cortical thickness (controlling for age, education, and medication)

Cortical Thickness	PANNS Positive			PANNS Negative		
	r	$P_{unadjusted}$	P_{FDR}	*r*	$P_{unadjusted}$	P_{FDR}
Right Superior Frontal	-0.165	0.204	0.407	-0.201	0.120	0.262
Left Superior Frontal	-0.157	0.226	0.407	-0.168	0.196	0.262
Middle Frontal	-0.218	0.091	0.273	-0.217	0.094	0.262
Inferior Frontal	-0.304	0.017	0.153	-0.183	0.157	0.262
Left Superior Temporal	-0.262	0.041	0.185	-0.162	0.211	0.262
Right Superior Temporal	-0.142	0.275	0.413	-0.165	0.204	0.262
Middle Temporal	-0.119	0.360	0.463	-0.214	0.098	0.262
Right Transverse Temporal	-0.042	0.747	0.747	-0.155	0.233	0.262
Left Transverse Temporal	-0.102	0.433	0.487	0.073	0.575	0.575

**FDR corrected p<0.05

Abbreviations. SZH, Schizophrenia

Supplementary Table 5.
Cortical Thickness, group comparisons (ANCOVA, Age, and Gender Covariates)

	Mean Thickness				
**Regions**	**HC**	**SZH**	***F***	$P_{unadjusted}$	P_{FDR}
Right Lateral orbitofrontal	2.634	2.552	15.007**	<0.001	0.010
Right Medial orbitofrontal	2.432	2.378	5.764**	0.018	0.063
Right Paracentral	2.452	2.403	4.809	0.030	0.092
Right Precentral	2.558	2.479	12.601**	0.001	0.010
Right Frontal pole	2.681	2.625	2.373	0.126	0.213
Left Lateral orbitofrontal	2.679	2.608	14.952**	<0.001	0.010
Left Medial orbitofrontal	2.475	2.415	9.751**	0.002	0.011
Left Paracentral	2.364	2.330	2.767	0.098	0.206
Left Precentral	2.590	2.520	10.226**	0.002	0.011
Left Frontal pole	2.738	2.644	8.51**	0.004	0.018
Right Banks of the Superior Temporal Sulcus	2.572	2.565	0.067	0.796	0.838
Right Entorhinal	3.493	3.437	1.263	0.263	0.329
Right Fusiform	2.702	2.639	9.152**	0.003	0.015
Right Inferior Temporal	2.788	2.716	13.018**	<0.001	0.010
Right Parahippocampal	2.699	2.655	1.437	0.233	0.315
Right Temporal Pole	3.738	3.635	3.287	0.072	0.172
Left Banks of the Superior Temporal Sulcus	2.507	2.488	0.711	0.401	0.458
Left Entorhinal	3.411	3.384	0.445	0.506	0.547
Left Fusiform	2.701	2.667	3.546	0.062	0.165
Left Inferior Temporal	2.753	2.707	5.639**	0.019	0.063
Left Parahippocampal	2.767	2.706	2.426	0.122	0.213
Left Temporal Pole	3.713	3.641	2.768	0.098	0.206
Right Superior Parietal	2.176	2.145	2.560	0.112	0.213
Right Inferior Parietal	2.466	2.444	1.367	0.244	0.315
Right Supramarginal	2.558	2.505	7.588**	0.007	0.028
Right Postcentral	2.092	2.051	4.373	0.038	0.109
Right Precuneus	2.383	2.349	3.274	0.073	0.172
Left Superior Parietal	2.186	2.174	0.446	0.505	0.547
Left Inferior Parietal	2.425	2.408	1.070	0.303	0.367
Left Supramarginal	2.561	2.507	9.959**	0.002	0.011
Left Postcentral	2.098	2.076	1.406	0.238	0.315
Left Precuneus	2.354	2.328	1.838	0.177	0.272
Right Lateral Occipital	2.190	2.162	2.677	0.104	0.208
Right Lingual	1.978	1.962	0.789	0.376	0.442
Right Cuneus	1.877	1.843	2.287	0.133	0.213
Right Pericalcarine	1.627	1.627	0.000	0.983	0.983
Left Lateral Occipital	2.143	2.124	1.555	0.215	0.315
Left Lingual	1.960	1.933	2.303	0.131	0.213
Left Cuneus	1.860	1.835	1.371	0.244	0.315
Left Pericalcarine	1.618	1.620	0.010	0.922	0.946

**FDR corrected p<0.05
Abbreviations. HC, Healthy Controls; SZH, Schizophrenia

Supplementary Table 6.
Correlations Between MATRICS Domains and cortical thickness in SZH (controlling for age, education, and medication)

Cortical Thickness	**Processing Speed**			**Attention/Vigilance**			**Visual Learning**			**Composite Scores**		
	r	$P_{unadjusted}$	P_{FDR}	*r*	$P_{unadjusted}$	P_{FDR}	*r*	$P_{unadjusted}$	P_{FDR}	*r*	$P_{unadjusted}$	P_{FDR}
Right Superior Frontal	0.070	0.595	0.822	0.215	0.093	0.192	0.339	0.007	0.021	0.205	0.122	0.183
Left Superior Frontal	0.045	0.731	0.822	0.213	0.096	0.192	0.382	0.002	0.018	0.213	0.109	0.183
Middle Frontal	0.133	0.305	0.822	0.204	0.112	0.192	0.350	0.005	0.021	0.266	0.043	0.183
Inferior Frontal	0.111	0.395	0.822	0.110	0.395	0.493	0.264	0.038	0.086	0.247	0.062	0.183
Left Superior Temporal	0.080	0.541	0.822	0.217	0.090	0.192	0.202	0.115	0.173	0.222	0.094	0.183
Right Superior Temporal	0.051	0.697	0.822	0.196	0.128	0.192	0.190	0.140	0.180	0.134	0.316	0.356
Middle Temporal	0.046	0.727	0.822	0.100	0.438	0.493	0.239	0.061	0.110	0.147	0.270	0.347
Right Transverse Temporal	0.163	0.208	0.822	0.223	0.081	0.192	0.171	0.184	0.199	0.246	0.063	0.183
Left Transverse Temporal	-0.005	0.970	0.970	0.069	0.593	0.593	0.165	0.199	0.199	0.042	0.756	0.756

**FDR corrected p<0.05
Abbreviations. SZH, Schizophrenia

Supplementary Table 7.
Correlations Between MATRICS Domains and cortical thickness in SZH (controlling for age, education, and medication)

Cortical Thickness	**Working Memory**			**Reasoning Problem Solving**			**Verbal Learning**		
	r	$P_{unadjusted}$	P_{FDR}	*r*	$P_{unadjusted}$	P_{FDR}	*r*	$P_{unadjusted}$	P_{FDR}
Right Superior Frontal	0.033	0.798	0.898	0.097	0.459	0.626	0.048	0.710	0.867
Left Superior Frontal	0.068	0.600	0.898	0.125	0.343	0.617	0.052	0.688	0.867
Middle Frontal	0.139	0.280	0.898	0.230	0.077	0.276	0.022	0.867	0.867
Inferior Frontal	0.047	0.716	0.898	0.219	0.092	0.276	-0.075	0.562	0.867
Left Superior Temporal	0.063	0.625	0.898	0.162	0.215	0.484	-0.090	0.489	0.867
Right Superior Temporal	-0.043	0.740	0.898	0.092	0.487	0.626	-0.084	0.518	0.867
Middle Temporal	-0.004	0.975	0.975	0.051	0.697	0.784	-0.028	0.826	0.867
Right Transverse Temporal	0.052	0.686	0.898	0.241	0.063	0.276	0.120	0.351	0.867
Left Transverse Temporal	-0.048	0.712	0.898	0.006	0.965	0.965	-0.073	0.571	0.867

**FDR corrected p<0.05
Abbreviations. SZH, Schizophrenia
